# A Giant Vocal Cord Polyp Mimics Asthma Attack

**DOI:** 10.5811/cpcem.2018.7.38347

**Published:** 2018-08-15

**Authors:** Yasuyuki Chida, Ryota Inokuchi, Yoshibumi Kumada, Kazuaki Shinohara

**Affiliations:** * Ohta Nishinouchi Hospital, Department of Emergency and Critical Care Medicine, Koriyama, Fukushima, Japan; † The University of Tokyo Hospital, Department of Emergency and Critical Care Medicine, Bunkyo-ku, Tokyo, Japan

## CASE PRESENTATION

A 38-year-old Japanese housewife who was a heavy smoker was admitted to our hospital because of upper respiratory distress that developed half a year prior to admission. She had no past medical history. On admission, her vital signs were normal. Physical examination showed hoarseness, stridor, wheezing, and orthopnoea, but no swelling of the tonsils, thyroid, or lymph nodes. A computed tomography of the neck revealed an enlarged tumor (Image A).

## DIAGNOSIS

Laryngeal endoscopy revealed a giant vocal cord polyp (Image B). Direct laryngoscopic resection after tracheostomy with local anesthesia was performed, which resulted in improved symptoms. After a week, the tracheal fenestra was closed and she was discharged without complication.

**CPC-EM Capsule**

What do we already know about this clinical entity?

Vocal cord polyps are common lesions, and most are small; the common symptom is hoarseness.

What is the major impact of the image(s)?

Giant vocal cord polyps can mimic asthma and may cause critical airway obstruction leading to sudden death.

How might this improve emergency medicine practice?

Large vocal cord polyp can mimic asthma. Thus, physicians should consider the disease when patients present with a protracted upper respiratory distress history.

Vocal cord polyps are common lesions, with a reported lifetime prevalence of 1.31% to 16.9% of the population.^1^ Mechanical or chemical irritation caused by heavy smoking can result in vocal cord polyps.^2^ Most vocal cord polyps are small lesions; thus, the common symptom is hoarseness. Occasionally, larger vocal cord polyps causing partial upper airway obstruction can mimic asthma.^3^,^4^ However, giant vocal cord polyps may cause critical airway obstruction leading to sudden death.^5^

Documented patient informed consent and/or Institutional Review Board approval has been obtained and filed for publication of this case report.

## Figures and Tables

**Image:**
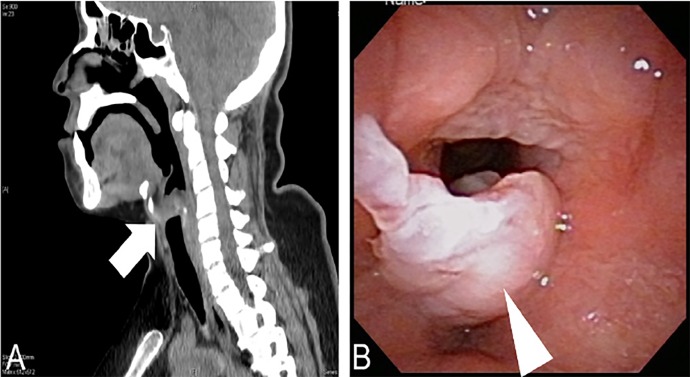
**A)** Sagittal cervical computed tomography showing a hypodense mass below the epiglottis (arrow).**B)** Laryngoscopy showing an elevated vocal polyp (arrowhead).
